# Precision or Personalized Medicine for Cancer Chemotherapy: Is there a Role for Herbal Medicine

**DOI:** 10.3390/molecules21070889

**Published:** 2016-07-07

**Authors:** Zhijun Wang, Xuefeng Liu, Rebecca Lucinda Ka Yan Ho, Christopher Wai Kei Lam, Moses Sing Sum Chow

**Affiliations:** 1 Center for Advancement of Drug Research and Evaluation, College of Pharmacy, Western University of Health Sciences, Pomona, CA 91766, USA; zwang@westernu.edu; 2 Department of Pathology and Center for Cell Reprogramming, Georgetown University Medical Center, Washington, DC 20057, USA; Xuefeng.Liu@georgetown.edu; 3 State Key Laboratory of Quality Research in Chinese Medicine, Macau Institute for Applied Research in Medicine and Health, Macau University of Science and Technology, Taipa, Macau, China; kyho@must.edu.mo

**Keywords:** precision medicine, personalized medicine, herbal medicine, resistant cancer

## Abstract

Although over 100 chemotherapeutic agents are currently available for the treatment of cancer patients, the overall long term clinical benefit is disappointing due to the lack of effectiveness or severe side effects from these agents. In order to improve the therapeutic outcome, a new approach called precision medicine or personalized medicine has been proposed and initiated by the U.S. National Institutes of Health. However, the limited availability of effective medications and the high cost are still the major barriers for many cancer patients. Thus alternative approaches such as herbal medicines could be a feasible and less costly option. Unfortunately, scientific evidence for the efficacy of a majority of herbal medicines is still lacking and their development to meet FDA approval or other regulatory agencies is a big challenge. However, herbal medicines may be able to play an important role in precision medicine or personalized medicine. This review will focus on the existing and future technologies that could speed the development of herbal products for treatment of resistant cancer in individual patients. Specifically, it will concentrate on reviewing the phenotypic (activity based) rather than genotypic (mechanism based) approach to develop herbal medicine useful for personalized cancer chemotherapy.

## 1. Introduction: Current Limitations of Cancer Chemotherapy

Although more than 100 chemotherapy drugs (National Cancer Institution, http://www.cancer.gov/about-cancer/treatment/drugs) including alkylating agents, antimetabolites, anti-tumor antibiotics, anthracyclines, topoisomerase inhibitors, mitotic inhibitors, corticosteroids, and other molecularly-targeted agents are available for cancer chemotherapy (either alone or in combination), the overall long term clinical benefit from these agents has been generally disappointing, and modern chemotherapy usually ends in failure due to either severe side effects or loss of effectiveness. One major reason for the loss of effectiveness is the development of chemoresistance [1,2,3,4,5]. To overcome such problem, a new approach called precision medicine or personalized medicine has been proposed and initiated by the National Institutes of Health (Bethesda, MD, USA). While the precision medicine approach is likely to yield more effective cancer treatment options in the future, the drug development cost is not expected to be reduced and thus the new drug cost for the patients will likely remain high. According to a recent report from the American Society of Clinical Oncology, the average cost to the patient of newly approved anticancer drugs is $10,000 per month and can be as high as $30,000/month [6]. Such cost is not only difficult to afford for a typical US citizen, it is unlikely to be affordable by more than 80% of the world population living in developing countries [7]. Thus an alternative approach, such as using herbal medicine, whether alone or in combination with conventional anti-cancer agents, could be a feasible and less costly option. But can herbal medicine meet the rigorously testing required and offer improved personalized cancer chemotherapy over conventional anticancer agents? If it is subjected to rigorous testing, can herbal medicine still be cost effective? We believe the answers to both questions can be an optimistic “yes”. This paper will first review the definition and perspectives of precision medicine versus personalized medicine relevant to research and clinical practice. We will then examine the existing and future technologies that could speed the development of herbal products for personalized cancer chemotherapy leading to enhanced efficacy and reduced cost for treatment of resistant cancer in individual patients. In order to speed the development that could rapidly lead to application in individual patients, this paper will concentrate in reviewing the phenotypic approach rather than genotypic/proteomic/metabolomics approaches to personalized cancer chemotherapy.

## 2. Precision Medicine vs. Personalized Medicine—Potential Application to Improved Cancer Chemotherapy

### 2.1. Definition of Precision Medicine and Personalized Medicine

Precision medicine refers to the tailoring of medical treatment to the individual characteristics of each patient. Similarly, personalized medicine usually refers to a medical approach that proposes the customization of healthcare—with medical decisions, practices, and/or products being tailored to the individual patient. Thus personalized medicine is often synonymous with precision medicine [8,9].

Since precision medicine relies on the comprehensive understanding of individual molecular profiles using genomic, proteomic, metabolomic, as well as bioinformatic approaches to obtain a thorough understanding of the correlation between the regulation of gene(s) (functional protein) and disease status, it depends largely on microarray and next generation sequencing (NGS) in obtaining the genetic information [10,11,12,13].

As genetic variations/mutations are often important factors relating to a particular disease as well as drug response, the first essential step for precision medicine would be the identification of such mutations in specific genes at multiple cell regulatory levels (such as genome, transcription, and epigenetics). Thus massive genetic screening of genetic variations is necessary in understanding the underlying mechanisms. Based on such information, the unique therapeutic strategy can be made for each individual patient having the genetic variation [14].

The regulation of genes can also occur at the stages of translation and post translation modification. In contrast to the genomic information which is relatively inherently static, dynamic processes of cells cannot be monitored using classical genomic approaches alone. Thus proteomic approaches have gained extensive development which can complement the molecular profiles. Proteomics will add additional information to precision medicine by monitoring the cellular function at the protein level and help to identify the quantitative biomarkers which can be essential for characterization of disease course and therapeutic response reliably [15,16].

Metabolomics is the qualitative identification and quantitative measurement of the dynamic multiparametric metabolic response in a biological system (such as cancer tissue and cells) under the condition of pathophysiological stimuli, genetic modification, or therapeutic treatment [17,18]. Endogenous metabolites can be determined by using GC-MS/LC-MS profiling [19,20]. Spectral data can be processed and annotated using commercially available database (NIST), and on-line database (HMDB). Based on such information the metabolite annotation and metabolomic biomarker can be selected.

Thus the “-omics” (genomics/proteomics/metabolomics) technology could enable researchers to further uncover the causative mechanisms or biomarkers and potentially optimize drug efficacy and safety.

### 2.2. Examples of Precision Medicine or Personalized Medicine in Cancer Chemotherapy

Precision medicine can involve a single drug or combination of drugs. A good example of single drug is pembrolizumab for metastatic non-small cell lung cancer (NSCLC) whose tumors express programmed death ligand 1 (PD-L1) and whose cancers progressed after platinum-based chemotherapy. When PD-L1 is activated, immune response is inhibited. Pembrolizumab targets PD-L1 and allows the host immune system to recognize and attack tumor cells (www.fda.gov).

A good example of combination therapy is pertuzumab in combination with trastuzumab and docetaxel for the treatment of human epidermal growth factor receptor 2 (HER2) positive metastatic breast cancer. Trastuzumab is an antibody that blocks the function of HER2, a protein produced by a specific gene with cancer-causing potential in HER2-positive metastatic breast cancer. Pertuzumab, another monoclonal antibody which binds to a different epitope on HER2 other than trastuzumab, can inhibit HER2 dimerization. The combination with pertuzumab has been shown to produce a median of 6.1 months longer survival time compared to without it [21].

### 2.3. Precision Medicine vs. Personalized Medicine—Perspectives in Drug Development and Practice

While precision medicine and personalized medicine are often synonymous in cancer chemotherapy, the initiating steps could be different in drug development versus practice. Precision medicine is usually a research initiative followed by practice, whereas personalized medicine often starts with an empiric practice followed by basic research (e.g., identification of biomarkers using genomic/proteomic/metabolomic technology) with subsequent implementation in clinical practice. Thus personalized medicine approach can begin with the initial step of empiric culture of cancer cells from patient’s tumor tissue followed by drug sensitivity testing using relevant techniques/models. Once an effective (high cytotoxicity to cancer cell) but safe (low cytotoxicity to normal cell) agent is found, individualization of its dose to achieve the desired target concentration can be implemented based on individual pharmacokinetics and pharmacodynamics. Additional research steps to identify genomic/proteomic/metabolomic biomarkers in response to drug therapy can be included. At present only a limited number of drugs have been developed to offer personalized cancer chemotherapy. The potential however is huge for future therapeutic agents including herbal medicines.

## 3. Technologies Relevant to Development of Herbal Medicine for Personalized Cancer Chemotherapy

To implement personalized cancer chemotherapy, a relevant pre-therapeutic diagnostic procedure such as biomarker identification or cancer cell culture and drug sensitivity determination is needed prior to proper therapy.

### 3.1. Biomarker Identification

The National Cancer Institute (NCI), in particular, defines biomarker as “a biological molecule found in blood, other body fluids, or tissues that is a sign of a normal or abnormal process, or of a condition or disease”. A biomarker can be used to assess the therapeutic response to chemotherapy in cancer patients.

The technologies of “-omics” have been widely used to identify the biomarkers of various cancers. Usually the identification of the biomarkers is initiated by collecting the biologically relevant samples which can be the biopsy tissues, patients’ blood and/or urine samples. The biomarker candidates can then be screened using proteomic techniques to identify the potential makers using appropriate protein array or mass spectrometry technologies. This can be incorporated with appropriate filtering criteria followed by validation of the candidate biomarkers using a large independent set of samples with targeted approaches such as SRM and ELISA. Pre-clinical assay development is followed by clinical validation. Final approval of the assay is obtained provided that the assay exceeds the current gold standard, is cost-effective, can easily be integrated into current clinical workflows, and improves patient management [22].

A good example is the quantitative proteomics approach for identifying mitochondrial proteins such as ACAT1 and MnSOD for clear cell renal cell carcinoma (ccRCC) [23]. Another example is the identification of eight new potential markers from formalin fixed paraffin embedded biopsy tissues by Metamark Genetics (Cambridge, MA, USA). Their proteomic profile/signature has been found to predict “favorable” versus “non-favorable” pathology of prostate cancer patients independently and may be used as alternatives for clinical decision [22,24,25]. Also the combination of prostate specific antigen (PSA) concentration with β-microseminoprotein (β-MSMB) level which was identified by using MALDI-MS profiling has increased the diagnostic sensitivity [26].

There are many other reports on identification and application of biomarkers using “-omics” technology. The detail review of the biomarker is beyond the scope of this paper. To apply to herbal medicines, the relationship in these biomarkers associated with herbal medicine needs to be established, then the biomarkers can be utilized as a useful monitoring tool for herbal medicine therapy.

### 3.2. Culture of Patient Cancer Cells and Determination of Drug Sensitivity

Another pretherapeutic diagnostic test is the cancer cell culture for determination of drug sensitivity. Over the past 50 years, the worldwide cancer research community has generated between 1000 and 1500 publically available cancer cell lines [27,28] and the use of such cell lines to determine drug sensitivity has been an accepted technique since the early 1980s [29,30]. While these conventional cancer cell lines have played an important role in understanding of tumor biology and high-throughput screening for drug development, such cancer cell lines generated with traditional methods usually fail to reflect the complex genotypes and phenotypes of the corresponding primary tumors due to in vitro selection and the accumulation of genetic and epigenetic alterations during passaging. Further, there is a lack of tumor derived extracellular matrix associated with these cell lines [31].

Thus, more relevant and better technique of culturing patient cancer cells for drug sensitivity testing can facilitate personalized medicine not only for conventional drugs but also for herbal products. While the present technology of culture and sensitivity is not matured enough for recommendation of its use as a diagnostic assay in clinical practice [32,33], a number of emerging techniques have provided significant progresses in the understanding of different aspects of cancer initiation, progression, metastasis and tumor microenvironment and can be useful for research and drug development, as described below.

#### 3.2.1. Collagen Gel Droplet Embedded Culture Drug Test (CD-DST)

This is a two-dimensional (2D) culture technique of patient cancer cells from tissue samples and it can be utilized for determination of drug sensitivity (e.g., using similar drug concentration exposure as that occurred in patients receiving the same drug). When comparing retrospectively the drug sensitivity from such in vitro determination has been found to correspond well to the therapeutic response in patients (sensitivity and specificity of 88.2% and 80.6% respectively with a predictive accuracy of about 84.1%) for a wide variety of cancer cells tested including lung, breast, gastric, esophagus [34]. Furthermore, the CD-DST drug sensitive results have been found to provide a better prediction to clinical response of gastric cancer, non-small cell lung cancer, or ovarian/uterine cancer when compared to those with drug resistant test results [35,36,37]. One importation limitation of this method is the need to obtain >10^4^ cells from biopsy samples for cell culture.

#### 3.2.2. Conditional Reprogramming (CR)

Liu et al. [38] developed a method which allows a rapid expansion of primary normal and tumor cells using combination of feeder cells and a Rho kinase inhibitor, Y-27632, termed Conditional Reprogramming (CR). CR seems to convert adult epithelial cells into a basal or stem-like state [39]. The induction of these conditionally reprogrammed cells (CRC) is reversible, and the removal of feeders and ROCK inhibitor, coupled with their placement in environments that mimic their native environment (Matrigel, air-liquid interface, and the renal capsule of immunodeficient mice) allows cells to differentiate normally. Importantly, the CR technology can generate 2 × 10^6^ cells in a week from small biopsies, and can generate cultures from cryopreserved tissue and from fewer than four viable cells. In one case report, a patient with lung tumor induced by a mutant HPV11 was treated with vorinostat based on the ex vivo response of their CR-derived tumor sample to this drug, and the patient showed a durable response to treatment [40]. A recent independent study utilized the CR method to initiate cultures from CT-guided lung biopsies from patients with non-small-cell lung cancer (NSCLC) who showed clinical resistance to targeted therapies. These cultures contain the same original driver mutations and maintain resistance to the single agent for which resistance was originally shown in patients. In addition, the use of CR technology has been shown to identify a novel combination of targeted therapies against MEK and ALK to combat resistance to single-agent ALK inhibition in ALK-mutant NSCLC. Such approach may well prove to be a suitable diagnostic test to identify therapeutic strategies for individual patients [41]

#### 3.2.3. Organoids and Organotypic Ex Vivo Technique

Another new technique is the organoids or 3D culture [42]. Organoids allow dissociated patient-derived cells to be expanded in a semi-solid extracellular matrix with growth-factor-enriched medium. Such an in vitro model has shown histologic, genomic, and phenotypic characteristics resembling that of the original tumor [43]. Organoids from patients with pancreatic, prostate and colon cancers have been developed and reported [42]. This technique however can require weeks to generate sufficient cells for drug testing, and the success rates of establishment are specific to the tissue of origin.

A recent improvement on the above is the organotypic ex vivo technique which incorporates both the tumor matrix proteins and autologous serum in the culturing of the patient’s cancer cells. Using this technique as well as an algorithm of responses from drug testing, an impressive predicted drug response rate (87%) has been obtained in 55 patients with head and neck squamous cell carcinoma with the testing being completed in about a week [44].

#### 3.2.4. Circulating Tumor Cells (CTC)

CTC or liquid biopsy is an attractive option for cancer cell culture and subsequent sensitivity testing. These cells are thought to be involved in metastasis and they usually die in the circulation due to circulatory shear stress or the loss of matrix related survival signals. The isolation and cultures of viable CTC are technically challenging, although there are a few reports with low success rates on certain types of metastatic tumor [45]. Its clinical utility is still being investigated at the present time [46].

#### 3.2.5. Patient Derived Xenograft (PDX) Model

The in vivo mouse cancer model, especially patient derived cancer xenograft (PDX), has been well accepted for investigation of mechanistic and new therapeutic strategies [47]. The PDX model involves directly implanting of fresh cancer tissue specimens into immunodeficient mice such as nude, SCID (severe combined immunodeficiency), and NSG (NOD scid gamma) mice. It is a more realistic preclinical model which can maintain the heterogeneous architecture and molecular characteristics of the original individual tumor as well as the microenvironment. The fresh tumor specimens can be grafted subcutaneously, orthotopically, and in subrenal capsule. It has been proven that the successful rate is highly dependent on the injection site. Subcutaneous injection is easy to implant. However, this site lacks vascularization and may result in loss of cancer subpopulations. The orthotopic model can best mimic the microenvironment similar to that of the original cancer. However, a complex surgical procedure is required for successful implantation. In addition, the orthotopic site has a limited capacity. Recently, subrenal capsule was found to be a more feasible site for prostate cancer model [48].

The PDX models have been used for prostate cancer and ovarian cancer research [49,50]. While the PDX model has a lot of advantages over the traditional xenograft model, its duration is very long (ranges from few months to years) and is not suitable for routine laboratory evaluation associated with clinical practice. It is more suitable for understanding mechanisms and therapeutic strategies and thus can be very helpful in the initial proof of concept for developing a new product or treatment strategy.

#### 3.2.6. Appraisal of the Cell Culture and Sensitivity Techniques for Herbal Product Evaluation

Among the currently available techniques as described above, the organotypic ex vivo test reported by Majumder et al. [44] appears to be the most useful. However, its technique is complicated compared to other in vitro tests. The CRC technique appears to be the simplest and fastest test available for determining cytotoxicity of various drugs including herbal compounds. The cells can be cultured in regular cell culture flask or plate. Afterwards, cytotoxicity of the compounds can be tested. The cell viability can also be tested by fluorescent and colorimetric cell viability assays (including tetrazolium-based assay (MTT), MTS cell proliferation assay, and sulforhodamine B assay) [51]. More recently, a technique for real time monitoring cell viability has been developed by detection of luminescent signal generate from the specific luciferase. If positive results are observed with a given compound, the bio-markers can be further evaluated using real time PCR, western blot or other immunological methods. Such biomarkers can further refine the clinical application when implementing the personalized cancer chemotherapy in the clinical practice [40].

A potential issue about activity of herbal medicines is the possibility of the presence of pan-assay interference compounds (PAINS) which can “function as reactive chemicals rather than discriminating drugs” for a specific target. [52,53]. However in resistant cancer, there are likely multiple targets. Will PAINS be actually beneficial for multiple targets? Further research is needed to investigate the relevance of PAINS and herbal therapy. Regardless, what is important is the empiric cytotoxic effect of the herbal product (with or without combination with the conventional anticancer agent) in resistant cancer and the safety of the herb.

### 3.3. Pharmacokinetic Approach for Optimal Dosing to Achieve Efficacy and Safety

Following the cancer cell culture and determination of drug sensitivity, dose optimization for achieving an effective and safe drug concentration in the patient is also an important step for personalized cancer chemotherapy. Pharmacokinetics (PK) describes the relationship of dosage to drug concentration in body fluids (e.g., plasma), while pharmacodynamics (PD) describes the relationship of drug concentration to the effect. Since the desirable dosage of an anticancer agent should ideally possess high cytotoxicity to the cancer cell but low cytotoxicity to the normal cell, a relevant PK/PD-model can be extremely useful to achieve this goal.

The simplest PK/PD model to use is the linear or log-linear model. An example of its application is for mitotane, an antineoplastic drug used in the treatment of adrenocortical carcinoma. Its minimum effective plasma concentration has been found to be about 14 μg/mL. Because it has a long elimination half-life (18–159 days), the accumulation of plasma concentration is relative slow and thus the onset of therapeutic effect is quite delayed (weeks or even months). A pharmacokinetic model has been established which enabled clinicians to adjust dosing based on a target drug exposure and facilitate personalized therapy with a coefficient of variation of 14%. With the aid of such model, the dose regimen can be adapted based on individual plasma level measurements in prospective setting, which makes it possible to improve the clinical management of mitotane treatment [54].

For predicting or simulating the drug response in cancer chemotherapy, we should also consider the heterogeneous nature of tumor tissues. To address cell heterogeneity, a multi-scale modeling approach has been proposed. A physiologic PK modeling to integrate the data obtained from in vitro U87 glioma cells, in vivo mice study and cancer patients for the brain disposition of a compound called temozolomide has been described with the DNA adducts serving as the marker for the PD model. This multiscale protocol may be further used for temozolomide PK-PD modeling in various cell populations, thus providing a critical tool to personalize temozolomide based chemotherapy on a cell-type-specific approach [55].

Since cancer chemotherapy often involves drug combination. A unified approach to optimize multidrug chemotherapy using a pharmacokinetic enhanced pharmacodynamic model has been developed. This model is based on the vascular endothelial growth factor receptor (VEGFR) signaling system characterized by ligand-receptor interactions, enzyme recruitment (Grb2-Sos, phospholipase Cγ (PLCγ), and phosphoinositide-3 kinase (PI3K)), and downstream mitogen-activated protein kinase and Akt cascade activation. Drugs targeting these mechanisms (a VEGF inhibitor, a PI3K inhibitor, a PLCγ inhibitor, and a mitogen-activated protein kinase inhibitor) and sunitinib can provide input to optimization-based control analyses. This method can capture the complexities of drug action, tailor cancer chemotherapy, and empower personalized medicine [56].

With more discoveries of genomic/proteomic/metabolomic markers which are being validated for cancer treatment, these markers can serve as the target in PKPD model. For example, the physiologically based PK modeling can simulate the drug disposition in tumor tissues in addition to the normal organs. By linking the drug exposure at the action site (tumor) to response (biomarkers), it is possible to evaluate the dynamic changes in the tumor cells.

Of the various PKPD models, a simple model such as the linear or log-linear model is more practical to empirically generate a desirable dosage to achieve a targeted therapeutic effect. Since the population pharmacokinetics is often already known for a given compound, a simple model is likely to be suitable for rapid simulation of dose concentration relationship of the active drug or active herbal component.

In individualized dosing, individual pharmacokinetics of the active drug can be first determined, then an individualized effective dose generated to achieve a targeted plasma concentration, similar to individualized dosing of aminoglycosides that has been established in the past, by using appropriate pharmacokinetic models [57,58,59]. However, the application of pharmacokinetics to individualization of herbal extracts (with multiple components) is difficult if not impossible. The identification of a major active component can be helpful to partially describe the dose-concentration-effect relationship. The conventional approaches to determine the effective and safe dose of the herbal extract including maximum tolerated dose of the herbal product in animals, existing human use experience, and dose ranging studies in human subjects can all offer useful consideration in deciding a suitable dose for the herbal product. In addition, information on the biomarker effect in relation to herbal product dosage will be especially useful.

## 4. Special Features of Herbal Medicine for Personalized Cancer Chemotherapy—Implications for Future Therapeutic Advancement

Precision medicine is often linked to the concept of targeting one mechanism with one specific compound. While this approach can be effective in certain single gene inherited diseases, there is no single target involvement in many other diseases, especially with resistant cancer. Consequently, in the latter situation, targeting more than one mechanisms would be more “precise”, since the single target approach has not been effective for long term efficacy. This is where the role of herbal medicines can be of benefit. The herbs contain multiple active components which are likely to target multiple mechanisms [60]. Recently at least two unique cytotoxic effects have been observed from certain herbs at low concentrations. These are the chemosensitizing effect (CE) and collateral sensitivity (CS). They may offer special utility in resistant cancer and are described below.

### 4.1. Chemosensitizing Effect (CE)

Based on observations in in vitro resistant cell lines and patient derived resistant cancer cells as well as animal studies, certain herbal extracts such as *Tripterygium wilfordii*, *Coptis* rhizome and *Rhei Rhizoma*, or their active single chemical components are capable of inducing a potent CE [3]. CE is the ability of a low concentration of herbal extract or its active component (e.g., using a half or one-quarter of IC_50_ concentration) capable of reversing anticancer drug resistance when combined with a particular anticancer drug which the cancer cell has already developed resistance. Some resistant cancer cells (such as PC3-TxR, a prostate cancer cells resistant to docetaxel) are known to be capable of over-expressing P-glycoprotein (P-gp), an efflux pump capable of pumping the active drug from intracellular site to extracellular site and thus decrease drug intracellular concentration. A well-known mechanism of CE from the herb *Tripterygium wilfordii* is believed to be the suppression of P-gp transporter in addition to several other mechanisms.

### 4.2. Collateral Sensitivity (CS) of Herbs

**Figure 1:**
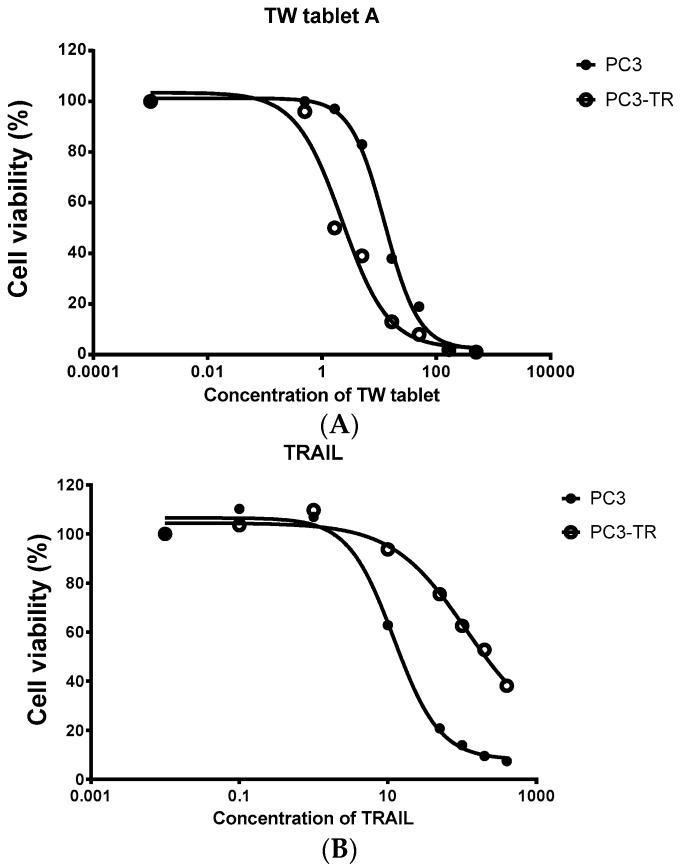
Cell viability (measured from triplicate samples) of a prostate cancer cell line (PC3) and its TRAIL resistant cell line (PC3-TR) treated with the extract of *Tripterygium wilfordii* (TW) tablet. (**A**) The cytotoxicity of TW tablets on PC3-TR was much more potent than PC3 (IC_50_ of 2.3 vs. 12.6 μg/mL for PC3-TR and PC3 respectively); (**B**) PC3-TR showed resistance to TRAIL (IC_50_ of 512 vs. 18 ng/mL for PC3-TR and PC3 respectively).

**Figure 2:**
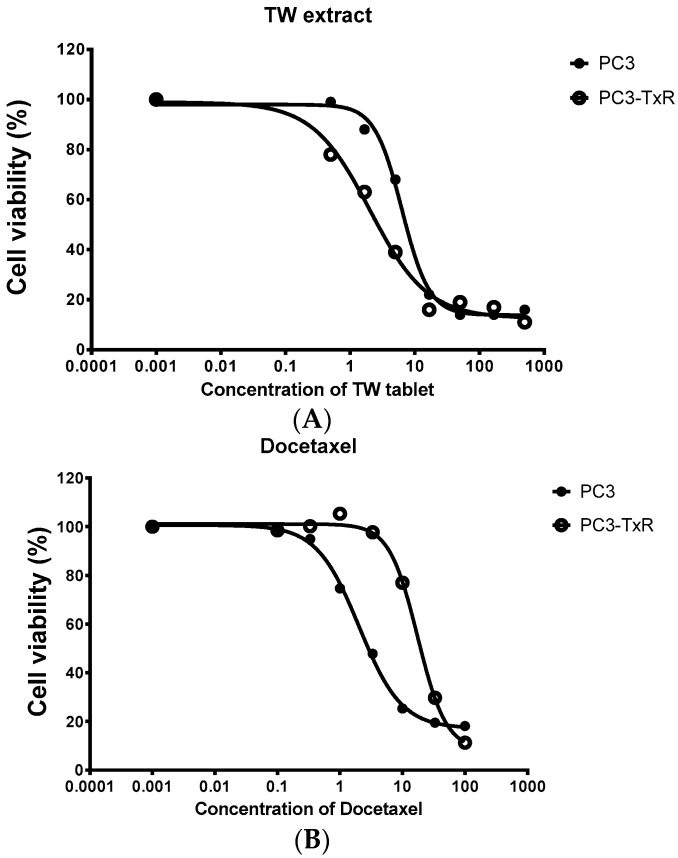
Cell viability (measured from triplicate samples) of a prostate cancer cell line (PC3) and its Dtx resistant cell line (PC3-TxR) treated with the extract of *Tripterygium wilfordii* (TW) extract. (**A**) The cytotoxicity of TW tablets on PC3-TxR was much more potent than PC3 (IC_50_ of 1.9 vs. 6.3 μg/mL for PC3-TxR and PC3 respectively); (**B**) PC3-TxR showed resistance to TRAIL (IC_50_ of 28.2 vs. 3.4 ng/mL for PC3-TxR and PC3 respectively).

CS is the ability of the herbal extract or compound to selectively inhibit the growth or kill the resistant cells more than the non-resistant parent cells. The mechanism is believed to be related to inhibition of anti-oxidant effect [61,62]. Certain flavonoids, verapamil, tiopronin and related compounds as well as extracts of *Tripterygium wilfordii* have been reported to significantly exert such effect in different resistant prostate cancer cell lines, as well as conditional reprogrammed cells from patients with breast, prostate and renal cell carcinoma [61,62]. In our preliminary test of collateral sensitivity of herbal products, the extracts of several commercially available *Tripterygium wilfordii* tablets (obtained from a Chinese medicine store in Shenzhen, China) were found more toxic in the prostate cancer cells resistant to TRAIL (PC3-TR) than its parent cell line (PC3) (See Figure 1 for one such product). In addition, a *Tripterygium wilfordii* extract was also shown significant collateral sensitivity in prostate cancer cells resistant to docetaxel (PC3-TxR) than its parent cell line (PC3) (see Figure 2). This could be an exciting area of herbal research. The combined CE and CS effects by herbal medicines could be an exciting potential to retard the development of chemoresistance, as based on clinical experience in antimicrobial chemotherapy and treatment of HIV infections [63,64]. Such activities may pave the way for further investigations to achieve enhanced benefit of herbal medicine in resistant cancer, which is a major problem at present.

## 5. Perspective and Future Direction of Herbal Medicine—Is there a Role for Personalized Medicine in Cancer Chemotherapy

While precision or personalized medicine involves the use of unique characteristics of a patient’s disease status (based on certain biomarkers or genomic/proteomic/metabolomic changes), this basic principle appears to share the same concept as the herbal medicine practice or Traditional Chinese Medicine (TCM) practice in China. In TCM practice, it has been well recognized that the practice is to provide an individualized therapeutic prescription based on an individual patient’s health condition. A typical TCM prescription for a given patient usually consists of a handful of ingredients mixed in a given ratio. Some of these ingredients are referred to as efficacy-enhancing ingredients which are empirically determined.

In this new era of precision medicine, genomic/proteomic/metabolomic markers may help to understand the mechanisms of enhancing effects of various ingredients of TCM prescriptions, e.g., by using profile (finger printing) of the herbs at “-omics” levels. (For example, tanshinone IIA has been shown to target P53 and AKT [65]. Aloe-emodin has been shown to inhibit H460 (non-small lung cancer cell line) by increasing the level of HSP70, 150-kD oxygen-regulated protein and protein disulfide isomerase using proteomics [66]. Ginsenoside Rg3 has been shown target the apoptosis associated proteins such as Rho GDP dissociation inhibitor, tropomyosin 1 and annexin V and glutathione s-transferase pi-1 [67]. Kampo-derived natural products have been shown to target a panel of genes related to transcriptional processes and nucleic acid interactions [68]). The biomarkers from these “-omics” technologies can help TCM to build a stronger evidence-based practice for personalized medicine, and may eventually not only improve cancer chemotherapy but also global health [69].

At present, herbal medicine has already been shown to prevent tumorigenesis, attenuating toxicity and enhancing efficacy as well as reducing tumor recurrence and metastasis [60]. These empiric results are encouraging and may be related to CE and CS effects from the herbs. However further research is needed to demonstrate the benefit from these 2 cytotoxic effects in resistant cancer cells. The fact that 49% of all existing anticancer drugs are either natural products (primarily herbal products) or their derivatives [70], the potential of applying these new cytotoxic actions from the herbal products to overcome resistance is highly attractive and may offer new hope to improve the low success rate (5%) for oncology drug development in the past [71]. While developing herbal product to achieve approval by FDA for resistant cancer with biomarker identification is likely a long process, its application using the personalized medicine approach can be tested as N = 1 trials to investigate the benefit of certain specific herbal product in resistant cancer. This can be initiated by empiric culturing resistant cancer cells from individual patient and determining sensitivity of herbal product followed by individualized dosing of the active herb (by utilizing the pharmacokinetics of its major active components). As many of these herbs have already been exposed to human subjects, such an approach should be feasible (after proper patient informed consent) and may offer a more timely therapeutic benefit to many individual patients who are suffering from chemotherapy resistant to conventional drugs.

## References

[1] Longley D.B., Johnston P.G. (2005). Molecular mechanisms of drug resistance. J. Pathol..

[2] Thomas H., Coley H.M. (2003). Overcoming multidrug resistance in cancer: An update on the clinical strategy of inhibiting p-glycoprotein. Cancer Control.

[3] Wang Z., Xie C., Huang Y., Lam C., Chow M. (2014). Overcoming chemotherapy resistance with herbal medicines: Past, present and future perspectives. Phytochem. Rev..

[4] Rottenberg S., Borst P. (2012). Drug resistance in the mouse cancer clinic. Drug Resist. Updates.

[5] Hamilton G., Rath B. (2014). A short update on cancer chemoresistance. Wien. Med. Wochenschr..

[6] Schnipper L.E., Davidson N.E., Wollins D.S., Tyne C., Blayney D.W., Blum D., Dicker A.P., Ganz P.A., Hoverman J.R., Langdon R. (2015). American Society of Clinical Oncology statement: A conceptual framework to assess the value of cancer treatment options. J. Clin. Oncol..

[7] Prasad S., Tyagi A. (2015). Traditional Medicine: The Goldmine for Modern Drugs. Adv. Tech. Biol. Med..

[8] Roden D.M., Tyndale R.F. (2013). Genomic medicine, precision medicine, personalized medicine: What’s in a name?. Clin. Pharmacol. Ther..

[9] Redekop W.K., Mladsi D. (2013). The faces of personalized medicine: A framework for understanding its meaning and scope. Value Health.

[10] Dong L., Wang W., Li A., Kansal R., Chen Y., Chen H., Li X. (2015). Clinical Next Generation Sequencing for Precision Medicine in Cancer. Curr. Genom..

[11] Zaneveld J., Wang F., Wang X., Chen R. (2013). Dawn of ocular gene therapy: Implications for molecular diagnosis in retinal disease. Sci. China Life Sci..

[12] Guo Y., Shi L., Hong H., Su Z., Fuscoe J., Ning B. (2013). Studies on abacavir-induced hypersensitivity reaction: A successful example of translation of pharmacogenetics to personalized medicine. Sci. China Life Sci..

[13] Vizirianakis I.S. (2002). Pharmaceutical education in the wake of genomic technologies for drug development and personalized medicine. Eur. J. Pharm. Sci..

[14] Chen G., Shi T. (2013). Next-generation sequencing technologies for personalized medicine: Promising but challenging. Sci. China. Life Sci..

[15] Mesri M. (2014). Advances in Proteomic Technologies and Its Contribution to the Field of Cancer. Adv. Med..

[16] Eckhard U., Marino G., Butler G.S., Overall C.M. (2016). Positional proteomics in the era of the human proteome project on the doorstep of precision medicine. Biochimie.

[17] Yu K.-H., Snyder M. (2016). Omics profiling in precision oncology. Mol. Cell. Proteom..

[18] Klement G.L., Arkun K., Valik D., Roffidal T., Hashemi A., Klement C., Carmassi P., Rietman E., Slaby O., Mazanek P. (2016). Future paradigms for precision oncology. Oncotarget.

[19] Pan L., Qiu Y., Chen T., Lin J., Chi Y., Su M., Zhao A., Jia W. (2010). An optimized procedure for metabonomic analysis of rat liver tissue using gas chromatography/time-of-flight mass spectrometry. J. Pharm. Biomed. Anal..

[20] Fordahl S., Cooney P., Qiu Y., Xie G., Jia W., Erikson K.M. (2012). Waterborne manganese exposure alters plasma, brain, and liver metabolites accompanied by changes in stereotypic behaviors. Neurotoxicol. Teratol..

[21] Baselga J., Cortés J., Kim S.-B., Im S.-A., Hegg R., Im Y.-H., Roman L., Pedrini J.L., Pienkowski T., Knott A. (2012). Pertuzumab plus Trastuzumab plus Docetaxel for Metastatic Breast Cancer. N. Engl. J. Med..

[22] Di Meo A., Pasic M.D., Yousef G.M. (2016). Proteomics and peptidomics: Moving toward precision medicine in urological malignancies. Oncotarget.

[23] Zhao Z., Wu F., Ding S., Sun L., Liu Z., Ding K., Lu J. (2015). Label-free quantitative proteomic analysis reveals potential biomarkers and pathways in renal cell carcinoma. Tumor Biol..

[24] Blume-Jensen P., Berman D.M., Rimm D.L., Shipitsin M., Putzi M., Nifong T.P., Small C., Choudhury S., Capela T., Coupal L. (2015). Development and Clinical Validation of an in situ Biopsy-Based Multimarker Assay for Risk Stratification in Prostate Cancer. Am. Assoc. Cancer Res..

[25] Shipitsin M., Small C., Choudhury S., Giladi E., Friedlander S., Nardone J., Hussain S., Hurley A.D., Ernst C., Huang Y.E. (2014). Identification of proteomic biomarkers predicting prostate cancer aggressiveness and lethality despite biopsy-sampling error. Br. J. Cancer.

[26] Flatley B., Wilmott K.G., Malone P., Cramer R. (2014). MALDI MS profiling of post-DRE urine samples highlights the potential of β-microseminoprotein as a marker for prostatic diseases. Prostate.

[27] Boehm J.S., Golub T.R. (2015). An ecosystem of cancer cell line factories to support a cancer dependency map. Nat. Rev. Genet..

[28] Barretina J., Caponigro G., Stransky N., Venkatesan K., Margolin A.A., Kim S., Wilson C.J., Lehar J., Kryukov G.V., Sonkin D. (2012). The Cancer Cell Line Encyclopedia enables predictive modelling of anticancer drug sensitivity. Nature.

[29] Frei E. (1982). The National Cancer Chemotherapy Program. Science.

[30] Venditti J.M. (1983). The National Cancer Institute antitumor drug discovery program, current and future perspectives: A commentary. Cancer Treat. Rep..

[31] Genovese L., Zawada L., Tosoni A., Ferri A., Zerbi P., Allevi R., Nebuloni M., Alfano M. (2014). Cellular localization, invasion, and turnover are differently influenced by healthy and tumor-derived extracellular matrix. Tissue Eng. Part A.

[32] Schrag D., Garewal H.S., Burstein H.J., Samson D.J., Von Hoff D.D., Somerfield M.R. (2004). American Society of Clinical Oncology Technology Assessment: Chemotherapy sensitivity and resistance assays. J. Clin. Oncol..

[33] Burstein H.J., Mangu P.B., Somerfield M.R., Schrag D., Samson D., Holt L., Zelman D., Ajani J.A. (2011). American Society of Clinical Oncology clinical practice guideline update on the use of chemotherapy sensitivity and resistance assays. J. Clin. Oncol..

[34] Kobayashi H. (2003). Development of a new in vitro chemosensitivity test using collagen gel droplet embedded culture and image analysis for clinical usefulness. Recent Results Cancer Res. Fortschr. Krebsforsch. Progres Dans Les Recherches Sur Le Cancer.

[35] Naitoh H., Yamamoto H., Murata S., Kobayashi H., Inoue K., Tani T. (2014). Stratified phase II trial to establish the usefulness of the collagen gel droplet embedded culture-drug sensitivity test (CD-DST) for advanced gastric cancer. Gastric Cancer.

[36] Higashiyama M., Oda K., Okami J., Maeda J., Kodama K., Imamura F., Minamikawa K., Takano T., Kobayashi H. (2010). Prediction of chemotherapeutic effect on postoperative recurrence by in vitro anticancer drug sensitivity testing in non-small cell lung cancer patients. Lung Cancer.

[37] Nagai N., Minamikawa K., Mukai K., Hirata E., Komatsu M., Kobayashi H. (2005). Predicting the chemosensitivity of ovarian and uterine cancers with the collagen gel droplet culture drug-sensitivity test. Anti-Cancer Drugs.

[38] Liu X., Ory V., Chapman S., Yuan H., Albanese C., Kallakury B., Timofeeva O.A., Nealon C., Dakic A., Simic V. (2012). ROCK inhibitor and feeder cells induce the conditional reprogramming of epithelial cells. Am. J. Pathol..

[39] Suprynowicz F.A., Upadhyay G., Krawczyk E., Kramer S.C., Hebert J.D., Liu X., Yuan H., Cheluvaraju C., Clapp P.W., Boucher R.C. (2012). Conditionally reprogrammed cells represent a stem-like state of adult epithelial cells. Proc. Natl. Acad. Sci. USA.

[40] Yuan H., Myers S., Wang J., Zhou D., Woo J.A., Kallakury B., Ju A., Bazylewicz M., Carter Y.M., Albanese C. (2012). Use of reprogrammed cells to identify therapy for respiratory papillomatosis. N. Engl. J. Med..

[41] Crystal A.S., Shaw A.T., Sequist L.V., Friboulet L., Niederst M.J., Lockerman E.L., Frias R.L., Gainor J.F., Amzallag A., Greninger P. (2014). Patient-derived models of acquired resistance can identify effective drug combinations for cancer. Science.

[42] Gao D., Vela I., Sboner A., Iaquinta P.J., Karthaus W.R., Gopalan A., Dowling C., Wanjala J.N., Undvall E.A., Arora V.K. (2014). Organoid cultures derived from patients with advanced prostate cancer. Cell.

[43] Gao D., Chen Y. (2015). Organoid development in cancer genome discovery. Curr. Opin. Genet. Dev..

[44] Majumder B., Baraneedharan U., Thiyagarajan S., Radhakrishnan P., Narasimhan H., Dhandapani M., Brijwani N., Pinto D.D., Prasath A., Shanthappa B.U. (2015). Predicting clinical response to anticancer drugs using an ex vivo platform that captures tumour heterogeneity. Nat. Commun..

[45] Yu M., Bardia A., Aceto N., Bersani F., Madden M.W., Donaldson M.C., Desai R., Zhu H., Comaills V., Zheng Z. (2014). Ex vivo culture of circulating breast tumor cells for individualized testing of drug susceptibility. Science.

[46] Alix-Panabieres C., Pantel K. (2014). Challenges in circulating tumour cell research. Nat. Rev. Cancer.

[47] Friedman A.A., Letai A., Fisher D.E., Flaherty K.T. (2015). Precision medicine for cancer with next-generation functional diagnostics. Nat. Rev. Cancer.

[48] Wang Y., Wang J.X., Xue H., Lin D., Dong X., Gout P.W., Gao X., Pang J. (2015). Subrenal capsule grafting technology in human cancer modeling and translational cancer research. Differentiation.

[49] Scott C.L., Becker M.A., Haluska P., Samimi G. (2013). Patient-derived xenograft models to improve targeted therapy in epithelial ovarian cancer treatment. Front. Oncol..

[50] Lin D., Xue H., Wang Y., Wu R., Watahiki A., Dong X., Cheng H., Wyatt A.W., Collins C.C., Gout P.W. (2014). Next generation patient-derived prostate cancer xenograft models. Asian J. Androl..

[51] Van Tonder A., Joubert A.M., Cromarty A.D. (2015). Limitations of the 3-(4,5-dimethylthiazol-2-yl)-2,5-diphenyl-2*H*-tetrazolium bromide (MTT) assay when compared to three commonly used cell enumeration assays. BMC Res. Notes.

[52] Baell J.B., Holloway G.A. (2010). New Substructure Filters for Removal of Pan Assay Interference Compounds (PAINS) from Screening Libraries and for Their Exclusion in Bioassays. J. Med. Chem..

[53] Baell J., Walters M.A. (2014). Chemistry: Chemical con artists foil drug discovery. Nature.

[54] Kerkhofs T.M., Derijks L.J., Ettaieb H., Den Hartigh J., Neef K., Gelderblom H., Guchelaar H.-J., Haak H.R. (2015). Development of a pharmacokinetic model of mitotane: Toward personalized dosing in adrenocortical carcinoma. Ther. Drug Monit..

[55] Ballesta A., Zhou Q., Zhang X., Lv H., Gallo J. (2014). Multiscale Design of Cell-Type–Specific Pharmacokinetic/Pharmacodynamic Models for Personalized Medicine: Application to Temozolomide in Brain Tumors. CPT Pharmacomet. Syst. Pharmacol..

[56] Zhang X.Y., Birtwistle M., Gallo J. (2014). A General Network Pharmacodynamic Model–Based Design Pipeline for Customized Cancer Therapy Applied to the VEGFR Pathway. CPT Pharmacomet. Syst. Pharmacol..

[57] Sawchuk R.J., Zaske D.E. (1976). Pharmacokinetics of dosing regimens which utilize multiple intravenous infusions: Gentamicin in burn patients. J. Pharmacokinet. Biopharm..

[58] Platt D.R., Matthews S.J., Sevka M.J., Comer J.B., Quintiliani R., Cunha B.A., Nightingale C.H., Chow M.S. (1982). Comparison of four methods of predicting serum gentamicin concentrations in adult patients with impaired renal function. Clin. Pharm..

[59] Burton M.E., Chow M.S., Platt D.R., Day R.B., Brater D.C., Vasko M.R. (1986). Accuracy of Bayesian and Sawchuk-Zaske dosing methods for gentamicin. Clin. Pharm..

[60] Ling C.-Q., Yue X.-Q., Ling C. (2014). Three advantages of using traditional Chinese medicine to prevent and treat tumor. J. Integr. Med..

[61] Pluchino K.M., Hall M.D., Goldsborough A.S., Callaghan R., Gottesman M.M. (2012). Collateral sensitivity as a strategy against cancer multidrug resistance. Drug Resist. Updates.

[62] Hall M.D., Marshall T.S., Kwit A.D.T., Miller Jenkins L.M., Dulcey A.E., Madigan J.P., Pluchino K.M., Goldsborough A.S., Brimacombe K.R., Griffiths G.L. (2014). Inhibition of Glutathione Peroxidase Mediates the Collateral Sensitivity of Multidrug-resistant Cells to Tiopronin. J. Biol. Chem..

[63] Imamovic L., Sommer M.O. (2013). Use of collateral sensitivity networks to design drug cycling protocols that avoid resistance development. Sci. Transl. Med..

[64] Munck C., Gumpert H.K., Wallin A.I., Wang H.H., Sommer M.O. (2014). Prediction of resistance development against drug combinations by collateral responses to component drugs. Sci. Transl. Med..

[65] Lin L.L., Hsia C.R., Hsu C.L., Huang H.C., Juan H.F. (2015). Integrating transcriptomics and proteomics to show that tanshinone IIA suppresses cell growth by blocking glucose metabolism in gastric cancer cells. BMC Genom..

[66] Lao Y., Wang X., Xu N., Zhang H., Xu H. (2014). Application of proteomics to determine the mechanism of action of traditional Chinese medicine remedies. J. Ethnopharmacol..

[67] Lee S.Y., Kim G.T., Roh S.H., Song J.S., Kim H.J., Hong S.S., Kwon S.W., Park J.H. (2009). Proteomic analysis of the anti-cancer effect of 20*S*-ginsenoside Rg3 in human colon cancer cell lines. Biosci. Biotechnol. Biochem..

[68] Efferth T., Miyachi H., Bartsch H. (2007). Pharmacogenomics of a traditional Japanese herbal medicine (Kampo) for cancer therapy. Cancer Genom. Proteom..

[69] Yun H., Hou L., Song M., Wang Y., Zakus D., Wu L., Wang W. (2016). Genomics and Traditional Chinese Medicine: A New Driver for Novel Molecular-Targeted Personalized Medicine?. Curr. Pharmacogenomics Pers. Med..

[70] Newman D.J., Cragg G.M. (2016). Natural Products as Sources of New Drugs from 1981 to 2014. J. Natl. Prod..

[71] Kola I., Landis J. (2004). Can the pharmaceutical industry reduce attrition rates?. Nat. Rev. Drug Discov..

